# Oleic acid chlorohydrin, a new early biomarker for the prediction of acute pancreatitis severity in humans

**DOI:** 10.1186/s13613-017-0346-6

**Published:** 2018-01-09

**Authors:** Enrique de-Madaria, Xavier Molero, Laia Bonjoch, Josefina Casas, Karina Cárdenas-Jaén, Andrea Montenegro, Daniel Closa

**Affiliations:** 1 Pancreatic Unit, Department of Gastroenterology, Hospital General Universitario de Alicante, Instituto de Investigación Sanitaria y Biomédica de Alicante (ISABIAL - Fundación FISABIO), Alicante, Spain; 2 Exocrine Pancreatic Diseases Research Group, Hospital Universitari Vall d’Hebron, Institut de Recerca (VHIR), Universitat Autònoma de Barcelona, CIBEREHD, Barcelona, Spain; 3 Department of Experimental Pathology, IIBB-CSIC, IDIBAPS, c/Rosselló 161, 7°, 08036 Barcelona, Spain; 4 RUBAM, Department of Biomedicinal Chemistry, IQAC-CSIC, Barcelona, Spain

**Keywords:** Acute pancreatitis, Chlorohydrins, Inflammation, Fatty acids, SIRS

## Abstract

**Background:**

The early prediction of the severity of acute pancreatitis still represents a challenge for clinicians. Experimental studies have revealed the generation of specific halogenated lipids, in particular oleic acid chlorohydrin, in the early stages of acute pancreatitis. We hypothesized that the levels of circulating oleic acid chlorohydrin might be a useful early prognostic biomarker in acute pancreatitis in humans.

**Methods:**

In a prospective, multicenter cohort study, plasma samples collected within 24 h after presentation in the emergency room from 59 patients with acute pancreatitis and from 9 healthy subjects were assessed for oleic acid chlorohydrin levels.

**Results:**

Pancreatitis was mild in 30 patients, moderately severe in 16 and severe in 13. Oleic acid chlorohydrin levels within 24 h after presentation were significantly higher in patients that later progressed to moderate and severe acute pancreatitis. Using 7.49 nM as the cutoff point, oleic acid chlorohydrin distinguished mild from moderately severe-to-severe pancreatitis with high sensitivity/specificity (96.6/90.0%) and positive/negative predictive values (90.3/96.4%). Using 32.40 nM as the cutoff value sensitivity, specificity, positive and negative predictive values were all 100% for severe acute pancreatitis. It was found to be a better prognostic marker than BISAP score, hematocrit at 48 h, SIRS at admission, persistent SIRS or C-reactive protein at 48 h.

**Conclusions:**

Oleic acid chlorohydrin concentration in plasma is elevated in patients with acute pancreatitis on admission and correlates with a high degree with the final severity of the disease, indicating that it has potential to serve as an early prognostic marker for acute pancreatitis severity.

## Background

Acute pancreatitis (AP) is an abrupt inflammatory process of the pancreas that often involves peripancreatic tissues and distant organs [1] and is one of the most common causes of hospital admission due to gastrointestinal disease [2]. The severity of the disease is highly variable, ranging from a mild and self-limited form to a severe disease with local and systemic complications that may lead to multiorgan failure and death [3].

The early prediction of severity becomes a critical issue in the management of AP. When the prediction is accurate, it allows for proper patient stratification with clinical management implications: Patients predicted at risk of developing severe AP will get enhanced hemodynamic support and close monitoring aimed at prompt recognition of local or systemic complications that may require specific treatment. Unfortunately, our ability to identify with high accuracy those severe cases early in the course of the disease is still unsatisfactory. The current prediction of severity is mainly based on multifactorial scores (Ranson, Imrie, APACHE-II, BISAP, etc.) [4, 5]. However, some of them are cumbersome to calculate (APACHE-II, Ranson, Imrie) and, most importantly, these scoring systems have a limited accuracy for mortality or persistent organ failure (> 48 h) [5–8]. In addition, some reports indicate that these scoring systems are not superior to easier-to-determine biomarkers, such as blood urea nitrogen, C-reactive protein (CRP), hematocrit or creatinine [6]. In this scenario, a blood biomarker with improved accuracy in predicting severe AP (as compared to previously described predictors) would be a step forward toward better early management of patients with severe AP.

**Fig. 1:**
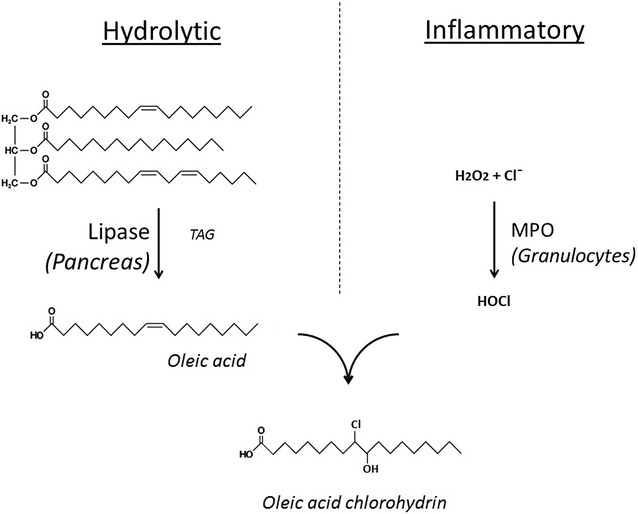
Generation of oleic acid chlorohydrin in acute pancreatitis. *TAG* triacylglycerol; *MPO* myeloperoxidase

In previous studies using an experimental model of taurocholate-induced acute pancreatitis in rats, we observed the generation and release to the bloodstream of specific halogenated lipids [9]. The infiltration and activation of polymorphonuclear neutrophils in the pancreas, but also in surrounding areas of adipose tissue, result in the generation of hypochlorous acid (HOCl) from hydrogen peroxide (H_2_O_2_) and chloride ions, in a reaction catalyzed by myeloperoxidase (MPO) [10]. The combination of high MPO and pancreatic lipase activity results in the generation and release of fatty acid chlorohydrins (Fig. 1), being the oleic acid chlorohydrin (OAC) the most abundant of them.

It is important to note that the generation of these halogenated fatty acids requires a combination of processes that are triggered during the development of pancreatitis, particularly when the enhanced inflammatory response acquires such characteristics that herald the progression to severe AP. This inflammatory response involves the recruitment of macrophages and neutrophils to the damaged pancreas and to the surrounding adipose tissue. These cells release active MPO in the areas where large amounts of pancreatic lipase catalyze the hydrolysis of triacylglycerols from adipocytes [11]. Therefore, fatty acid chlorohydrins (and in particular OAC) generation is fostered by the combined action of two enzymatic systems (myeloperoxidase and lipase) that are overactive in severe AP. Furthermore, OAC can be released to the bloodstream and its blood levels quantitated.

Experimental studies pointed to an interesting correlation between the levels of circulating OAC and the severity of acute pancreatitis [12]. However, the potential use of plasma OAC concentration as an early biomarker for severe AP in humans has not been evaluated yet. In this study, we assessed the effectiveness and feasibility of OAC in the early prediction of severity of acute pancreatitis in humans.

## Methods

The relationship between OAC plasma levels and outcome of AP was studied in a prospective cohort of consecutive patients from two medical centers: Hospital General Universitario de Alicante (Alicante, Spain) and Hospital Universitari Vall d’Hebron (Barcelona, Spain). The study protocol was approved by the ethic committee of both centers and followed the Declaration of Helsinki Ethical Principles for Medical Research Involving Human Subjects. The patients signed an informed consent before entering the study.

### Study population

Adult (≥ 18 years) patients with AP were prospectively recruited for 6 months. AP was defined according to the revised Atlanta classification. At least two of the following three features should be presented: (1) abdominal pain consistent with acute pancreatitis (acute onset of a persistent, severe, epigastric pain often radiating to the back); (2) serum lipase activity (or amylase activity) at least three times greater than the upper limit of normal; and (3) characteristic findings of acute pancreatitis on contrast-enhanced computed tomography (CECT) and less commonly on magnetic resonance imaging (MRI) or on transabdominal ultrasonography [13]. We excluded patients with recurrent acute pancreatitis, chronic pancreatitis, pancreatitis due to malignancy, pregnant patients, patients with time from onset of disease to presentation in the emergency room greater than 24 h and patients being hospitalized for more than 24 h at the time of recruitment.

### Variables

Local and systemic complications were defined according to the revised Atlanta classification [13], and systemic inflammatory response syndrome (SIRS) was defined according to usual criteria [14]. We compared the prognostic accuracy of OAC with other commonly used prognostic markers and scores: BISAP score [15], C-reactive protein at 48 h from presentation [16], presence of SIRS criteria at emergency room and presence of persistent SIRS (> 48 h) [17, 18] and hematocrit at emergency room higher than 44% [19]. Charlson comorbidity index was used to describe comorbidity [20].

Outcome variables were defined according to the revised Atlanta classification [13]: moderately severe-to-severe disease (presence of local or systemic complications in general), severe disease (presence of persistent organ failure) and in-hospital mortality.

### Blood samples

Blood samples were collected from each patient within 24 h after presentation in the emergency room, drawn into 5-ml heparin-treated tubes and centrifuged for 10 min at 1500×g. The plasma was then collected and stored at − 40 °C until analysis. Healthy controls were recruited from hospital staff. Assessment of OAC concentration was done blindly.

### Oleic acid chlorohydrin analysis by mass spectrometry

Samples were fortified with 18,18,18-d3-octadecanoic acid (20 nmol) and extracted with hexane (four volumes). The organic extract was evaporated and lipid extracts were derivatized with bis-trimethylsilyltrifluoroacetamide and injected the GC–MS system [21]. Endogenous levels of fatty acid chlorohydrins were thus quantified by selected ion monitoring (SIM).

Gas chromatography coupled to electron impact (70 eV) mass spectrometry was carried out as described [21] with minor modifications. Selected ions were those at *m*/*z* 344 and *m*/*z* 359 ([(M-CH3)^+^ and (M)^+^, trimethylsilyl (TMS) derivative of internal standard]; *m*/*z* 215 (TMS derivative of oleic acid chlorohydrin) and *m*/*z* 317 (common to the TMS derivatives of both the chlorohydrins of oleic and linoleic acids).

### Statistical methods

Results are expressed in mean (SD), median (*Q*1, *Q*3) or *n* (%). Normality was assessed by means of the Shapiro–Wilk test. Quantitative variables were compared with qualitative variables by means of the Student *t*/Mann–Whitney tests (2 categories) or ANOVA/Kruskal–Wallis tests (> 2 categories). Qualitative variables were compared by means of the Chi-square test, with Fisher’s correction when necessary. Receiver operating characteristics (ROC) curves were calculated for assessing the prognostic accuracy and for determining the best cutoff points. Sensitivity (Se), specificity (Sp) and positive/negative predictive values (PPV and NPV, respectively) were calculated. A *P* value less than 0.05 indicated statistical significance, but we applied the Bonferroni correction when multiple comparisons were performed. Statistical analyses were performed using SPSS 19.0 (SPSS, Inc., Chicago, Illinois, USA).

## Results

### Demographic, clinical and biochemical characteristics of patients

Fifty-nine patients and nine healthy subjects entered the study. Based on the revised Atlanta classification [13], 30 patients had mild AP, 16 developed moderately severe AP and 13 severe AP. As the two hospitals are transferal centers where they derived the most serious cases, the percentage of severe pancreatitis is relatively high. Patient’s basal clinical characteristics as well as outcomes according to severity are shown in Table 1. Gallstones were the main etiological factor, followed by alcohol abuse and endoscopic retrograde cholangiopancreatography (ERCP). Eight patients with severe AP died (5 of them with infected pancreatic necrosis). No significant differences were observed in age, gender or BMI > 30 between groups.

**Table 1:** Demographic characteristics and outcomes of the study patients according to severity

	Global sample (*n* = 59)	Mild (*n* = 30)	Moderately severe (*n* = 16)	Severe (*n* = 13)	*P*
Age (years)	64 (50.5–77)	62.5 (49.5, 72.3)	66.5 (53.5, 77.8)	77 (42, 81.5)	0.5
Female sex	33 (55.9%)	19 (63.3%)	9 (56.3%)	5 (38.5%)	0.3
Etiology					0.065
Biliary	24 (40.7%)	13 (43.3%)	7 (43.8%)	4 (30.8%)	
Alcohol	8 (13.6%)	1 (3.3%)	4 (25%)	3 (23.1%)	
Post-ERCP	7 (11.9%)	3 (10%)	2 (12.5%)	2 (15.4%)	
BMI > 30 kg/m^2^	14 (23.7%)	7 (23.3%)	3 (18.8%)	4 (30.8%)	0.749
BISAP score	1 (1, 2)	1 (0, 1.3)	2 (1, 3)	2 (1, 3)	0.005
Charlson index	0 (0, 1)	0 (0, 1)	1 (0, 2)	1 (2.5)	0.035
CRP at 48 h (mg/dL)	10.9 (3–24.2)	4.3 (1.8, 12.7)	19.8 (9.6, 28.4)	30.4 (23.2, 39.3)	<0.001
OAC (nM)	7 (2.3–22.6)	2.6 (1.3, 5)	14.7 (9.1, 22.6)	65.2 (42, 71.4)	<0.001
SIRS at ER	29 (49.2%)	9 (30%)	11 (68.8%)	9 (75%)	0.007
SIRS > 48 h	14 (23.7%)	0	3 (18.8%)	11 (84.6%)	<0.001
Pancreatic necrosis	9 (15.3%)	0	3 (18.8%)	6 (46.2%)	0.001
Peripancreatic necrosis	15 (25.4%)	0	9 (56.3%)	6 (46.2%)	<0.001
Infected necrosis	5 (8.5%)	0	0	5 (38.5%)	<0.001
Persistent organ failure	13 (22%)	0	0	13 (100%)	<0.001
Hospital stay (days)	12 (7–18.5)	8.5 (6, 12.3)	14 (11, 20)	32.5 (3.8, 52.3)	0.014
Mortality		0	0	8 (61.5%)	<0.001

Results in median (p25, p75) or *n* (%)

*BISAP* Bedside index of severity in acute pancreatitis, *SIRS* systemic inflammatory response syndrome, *OAC* oleic acid chlorohydrin, *CRP* C-reactive protein, *ERCP* endoscopic retrograde cholangiopancreatography, *ER* emergency room. Please note that persistent organ failure defines the severe category on the revised Atlanta classification

*P* statistical significance for the comparison between the mild, moderately severe and severe categories

### Oleic acid chlorohydrin levels in plasma

**Fig. 2:**
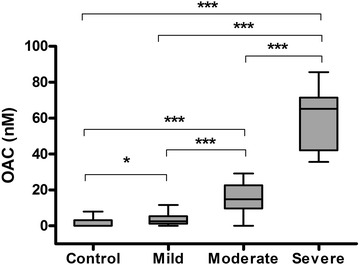
Oleic acid chlorohydrin levels (nM) measured on admission in plasma of patients with acute pancreatitis that finally evolved into different disease severities. *OAC* oleic acid chlorohydrin; *P < 0.05; ***P < 0.001. Level of significance according to Bonferroni correction: 0.0125

Median OAC plasma concentration raised as the severity of AP increased. Significant differences in plasma OAC levels were detected among the different group categories of AP studied: mild, moderately severe and severe (Fig. 2), with lower levels in the mild acute pancreatitis group (median 2.6 nM; 1.3–5) as compared with moderate (14.7 nM; 9.1–22.65) and severe (65.2 nM; 42–71.4) groups. OAC was significantly different in all the possible comparisons between controls, mild, moderately severe and severe categories (Fig. 2). In control samples, OAC was only detected in a few samples (0.0 nM; 0–3.1).

### Prognostic accuracy of OAC and other predictors

**Fig. 3:**
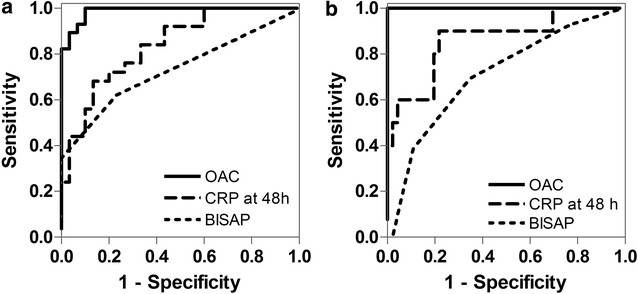
ROC curves of oleic acid chlorohydrin (OAC), C-reactive protein (CRP) and BISAP score for the prediction of acute pancreatitis severity. **a** ROC curve for the prediction of moderately severe-to-severe disease. **b** ROC curve for the prediction of severe disease. *OAC* oleic acid chlorohydrin. *CRP at 48* *h* C-reactive protein at 48 h from admission. *BISAP* bedside index of severity in acute pancreatitis

Regarding the prediction of moderately severe-to-severe AP, the ROC curves for OAC, BISAP score and CRP are shown in Fig. 3. OAC levels had a higher ROC AUC than BISAP score and CRP (Table 2). Using 7.49 nM as the cutoff point, OAC plasma concentration at admission could readily predict moderately severe or severe AP with a sensitivity of 96.6%, specificity of 90.0%, positive predictive value (PPV) of 90.3% and negative predictive value (NPV) of 96.4%. Sensitivity and NPV for OAC were higher than for other commonly used predictors (Table 3).

**Table 2:** Area under the receiver operating characteristics curve for the prediction of moderately severe-to-severe disease, severe disease (persistent organ failure) and mortality for oleic acid chlorohydrin, BISAP score and C-reactive protein

Outcome to predict	Predictor	AUC (95% CI)
Moderately severe-to-severe disease	OAC	0.954 (0.886–1)
	BISAP score	0.732 (0.601–0.863)
	CRP 48 h	0.837 (0.734–0.941)
Severe disease (persistent organ failure)	OAC	1
	BISAP score	0.709 (0.547–0.871)
	CRP 48 h	0.860 (0.720–1)
Mortality	OAC	0.931 (0.866–0.997)
	BISAP score	0.808 (0.668–0.948)
	CRP 48 h	0.772 (0.529–1)

*AUC* Area under the receiver operating characteristics curve, *CI* confidence intervals, *OAC* oleic acid chlorohydrin, *BISAP* Bedside index of severity in acute pancreatitis, *CRP 48* *h* C-reactive protein at 48 h from admission

**Table 3:** Prognostic accuracy of OAC for moderately severe-to-severe disease compared to other widely used predictors

Variable	Se	Sp	PPV	NPV
OAC ≥ 7.49 nM	96.6	90	90.3	96.4
SIRS at ER	71.4	70	69	72.4
Persistent SIRS	48.3	100	100	65.9
HTC at ER > 44%	44.8	66.7	56.5	55.6
BISAP ≥ 3	34.5	100	100	61.2
CRP ≥ 15 mg/dL at 48 h	68	80	73.9	75

*Se* Sensitivity, *SP* specificity, *PPV/NPV* positive/negative predictive values, *POF* persistent organ failure, *OAC* oleic acid chlorohydrin, *SIRS* systemic inflammatory response syndrome, *HTC* hematocrit, *BISAP* bedside index of severity in acute pancreatitis, *CRP* C-reactive protein

OAC was an excellent predictor of severe disease (and persistent organ failure), again associated with higher AUC (1) than BISAP score and CRP (Table 2; Fig. 3). Using 32.40 nM as the cutoff value, OAC predicted severe disease with 100% sensitivity, specificity, PPV and NPV. This accuracy was higher than any other predictor evaluated in this study (Table 4).

**Table 4:** Prognostic accuracy of OAC for severe disease (persistent organ failure) and mortality compared to other widely used predictors

Variable	Outcome to predict	Se	Sp	PPV	NPV
OAC ≥ 32.4 nM	Severe disease	100	100	100	100
	Mortality	100	90.2	61.5	100
SIRS at ER	Severe disease	75	56.5	31	89.7
	Mortality	85.7	54.9	20.7	96.6
Persistent SIRS	Severe disease	84.6	93.3	78.6	95.5
	Mortality	75	84	42.9	95.5
HTC at ER > 44%	Severe disease	38.5	60.9	21.7	77.8
	Mortality	25	58.8	8.7	83.3
BISAP ≥ 3	Severe disease	38.5	89.1	50	83.7
	Mortality	50	88.2	40	91.8
CRP ≥ 15 mg/dL at 48 h	Severe disease	90	68.9	39.1	96.9
	Mortality	80	62	17.4	96.9

*Se* Sensitivity, *SP* specificity, *PPV/NPV* positive/negative predictive values, *POF* persistent organ failure, *OAC* oleic acid chlorohydrin, *SIRS* systemic inflammatory response syndrome, *HTC* hematocrit, *BISAP* bedside index of severity in acute pancreatitis, *CRP* C-reactive protein

It is interesting to note that all of the above results did not change significantly when data from patients recruited at each participating hospital were processed independently, indicating that the test performed equally well at the two hospitals.

## Discussion

Early identification of severe forms of AP is essential for successful management of affected patients. Unfortunately, early reliable and sensitive predictive markers of severity are not at hand for application in clinical practice beyond popular multifactorial scores [22–24]. Moderately severe-to severe acute pancreatitis is believed to be the result of a multifactorial process that involves tissue necrosis, hydrolytic enzyme activation, release of inflammatory cytokines, generation of reactive oxygen species and synthesis of bioactive lipid mediators. In addition, some of these events take place in extra-pancreatic organs and none of them, by its own, is directly linked to the severity of the disease. Of note, pancreas-derived mediators that proved valuable for severity prediction in experimental animals have failed to predict the future course of pancreatitis when applied to human disease. The concomitant activation of different local and systemic processes explains the need for multifactorial scoring systems developed to predict the severity of AP, including Ranson criteria, APACHE-II or BISAP [5].

Fatty acid chlorohydrins are halogenated lipids generated by the action of hypochlorous acid on unsaturated fatty acids [10]. Although absent in control conditions, they can be released to the bloodstream during AP due to the combined effect of high lipase and MPO activities. Since this combination is characteristic of severe pancreatic/peripancreatic damage and enhanced systemic response, the presence of fatty acid chlorohydrins could only be expected to occur under the simultaneous activation of both hydrolytic and inflammatory processes that, when combined, are associated with the progression from mild to severe AP. Importantly, a number of pro-inflammatory activities have also been reported for fatty acid chlorohydrins, including the induction of P-selectin and the activation of macrophages [9, 25].

The value of lipid metabolites as indicators of increased severity of AP has been progressively recognized. There are also some clues that point to an active role of lipid mediators in the progression of mild to severe AP, in particular on the systemic effects that may lead to organ failure. Obesity or increased intra-abdominal fat is associated with severe AP, possibly through a mechanism related to the lipotoxic action of unsaturated fatty acids released by the effect of lipase on triglycerides of visceral adipocytes. In a series of elegant experiments, Navina et al. [26] demonstrated the pathogenic role of unsaturated fatty acids released during pancreatitis on inflammation, necrosis, multisystem organ failure and mortality. The measurement of chlorohydrins of fatty acids is a further step on this direction since the presence of these molecules indicates that, in addition to the effects of hydrolytic enzymes released by the pancreas, the inflammatory microenvironment required for the halogenation of fatty acids becomes relevant in distant organs. Even when these reactions are only relevant in local scenarios, the plasma levels of OAC are ultimately related to increased severity of AP and to multiorgan failure. The experimental study in rats indicates that release of OAC depends on the action of lipase following a kinetic similar to that observed for oleic acid, whereas its final course seems to be related to the hepatic uptake of free fatty acids [9]. Chlorinated fatty acids have been reported to be catabolized by hepatocytes through *ω*-oxidation and subsequent *β*-oxidation and their final products are rapidly excreted in urine [27]. Altogether it suggests that OAC could be a useful prognostic parameter but only in the initial stages of acute pancreatitis.

Our results in human patients confirm the expectations generated in experimental studies and show the value of OAC in predicting the course and outcome of AP. OAC was particularly accurate in predicting persistent organ failure and mortality, the most important outcome variables in AP. Our data reveal that levels of OAC measured on admission allow for discriminating the different degrees of severity to be developed on the progression of the disease in the long run, which should have a direct impact on the clinical management of these patients. According to our data, OAC concentrations higher than 32.4 nM are associated with severe AP, while a cutoff value of 7.5 distinguishes mild from moderate and severe disease.

Like many other studies focusing on research of prognostic biochemical markers, our study also has a number of methodological and technical limitations. The study includes patients from two hospitals, but, as expected, the number of patients in moderate and severe groups was significantly lower than in mild group. Therefore, out of 59 patients with AP only 13 had severe pancreatitis and 16 moderately severe. Although the results obtained allow to discriminate between these three groups with a good statistical level, there is no doubt that more studies with a much higher number of patients need to be done. Anyway, OAC was very accurate to detect severity and was compared with preexisting predictors of severity that were applied to the same sample showing a lower accuracy. On the other hand, the generation of OAC through the incorporation of the HOCl to the 9,10 double bond of oleic acid results in the formation of two isomeric chlorohydrins (the 9-chloro, 10-OTMS 18:0 chlorohydrin and the 9-OTMS, 10-chloro 18:0 chlorohydrin). These compounds are not separated by the GC column and, although this limitation may have little impact on the overall results of our study, we have to acknowledge that our data indicate the sum of the two isomers. In addition, it must be taken into account that, although the measure of OAC by gas chromatography/mass spectrometry is too complex and slow to be applied to clinical practice, ELISA tests similar to those for prostaglandins and other lipid metabolites can be easily developed allowing for much faster measurements of OAC. Availability of faster and easier assays methods will make easier to confirm our results in larger samples of patients. In this line, although these encouraging results have been obtained from two independent cohorts of patients from two different and geographically remote hospital centers, they should be validated in larger cohort studies performed by other groups. In case of similar results, OAC would be, by far, the most accurate early predictor of severe acute pancreatitis ever described.

## Conclusions

In summary, this study demonstrates that OAC is generated during AP, it can be measured in plasma, and its levels correlate with pancreatitis severity. Our findings indicate that oleic acid chlorohydrin is an accurate early prognostic biomarker to be used in patients with acute pancreatitis.

## Abbreviations

AP: acute pancreatitis
OAC: oleic acid chlorohydrin
CRP: C-reactive protein
ROC: receiver operating characteristics
BISAP: bedside index for severity in acute pancreatitis
SIRS: systemic inflammatory response syndrome
POF: persistent organ failure
